# Severe Thrombocytopenia in Pregnancy: Etiology, Management, and Outcomes Across Platelet Count Categories

**DOI:** 10.3390/jcm14228162

**Published:** 2025-11-18

**Authors:** Fırat Ersan, Verda Alpay, Barış Boza, Hakan Erenel

**Affiliations:** Department of Obstetrics and Gynecology, Division of Perinatology, Başakşehir Çam and Sakura City Hospital, 34490 Istanbul, Turkey; verda_alpay@yahoo.com (V.A.); barisboza@hotmail.com (B.B.); hakanerenel@yahoo.com (H.E.)

**Keywords:** thrombocytopenia, severe thrombocytopenia, immune thrombocytopenia, HELLP syndrome, perinatal outcomes, pregnancy

## Abstract

**Background/Objectives**: Severe thrombocytopenia (platelet count ≤ 50,000/µL) is a diagnostically heterogeneous condition during pregnancy, encompassing obstetric and non-obstetric etiologies that require distinct management approaches. The aims of this study were to determine the etiological distribution of severe thrombocytopenia during pregnancy and to evaluate its management strategies and perinatal outcomes in a tertiary perinatology center. **Methods**: This retrospective cohort study included 203 pregnant women with severe thrombocytopenia (platelet count ≤ 50,000/µL) stratified into three groups: Group A (30,000 < platelet count ≤ 50,000/μL, *n* = 123), Group B (10,000 < platelet count ≤ 30,000/μL, *n* = 54), and Group C (<10,000/µL, *n* = 26). Demographic characteristics, etiological diagnoses, treatment modalities, and perinatal outcomes were evaluated. **Results**: The etiological distribution varied significantly across severity groups (*p* = 0.001). HELLP (hemolysis, elevated liver enzymes, and low platelet count) syndrome was the most common cause overall (36.5%) and predominated in milder thrombocytopenia (Group A: 40.7%; Group B: 42.6%), whereas non-obstetric etiologies, such as immune thrombocytopenia (ITP), were significantly more frequent in Group C (57.7%). Treatment intensity increased with severity, with 79.7% of Group A requiring no intervention compared to only 26.9% of Group C (*p* = 0.001). Gestational age at delivery (median 37 weeks, *p* = 0.587) and birth weight (mean 2547 ± 968 g, *p* = 0.191) were comparable across severity groups. Minimum platelet count showed no significant correlation with delivery timing, birth weight, or hemoglobin decline. **Conclusions**: Severe thrombocytopenia in pregnancy exhibits distinct etiological patterns that vary according to platelet count severity. Favorable perinatal outcomes are achievable with appropriate diagnosis and management in specialized centers, underscoring the importance of comprehensive diagnostic evaluation rather than relying solely on platelet count thresholds for clinical decision-making.

## 1. Introduction

Thrombocytopenia, defined as a platelet count below 150.000/µL, complicates approximately 7–10% of all pregnancies and is the second most common hematological disorder during pregnancy after anemia [1]. Although most cases occur in the third trimester, this condition can arise at any time during gestation [2]. In pregnancy, the platelet count decreases predominantly due to abnormal platelet activation, increased immune-mediated destruction, or increased consumption, rather than impaired production [3].

Several conditions are associated with pregnancy-related thrombocytopenia. Some of these, including gestational thrombocytopenia (GT), hypertensive disorders such as preeclampsia, and HELLP (hemolysis, elevated liver enzymes, low platelet count) syndrome, are unique to pregnancy, whereas others, such as immune thrombocytopenia (ITP), systemic lupus erythematosus (SLE), or antiphospholipid antibodies syndrome (APLA), can occur independently of pregnancy [4,5]. GT, a benign, self-limiting disorder, accounts for nearly 75% of cases, followed by pregnancy-specific hypertensive disorders (15–20%), and ITP (3–4%) [4]. It is typically characterized by a mild reduction in platelet count (100.000–150.000/µL) and poses no significant risk to either the mother or the fetus [6]. However, thrombocytopenia can also be a sign of more severe underlying pathologies, making accurate diagnosis and management a critical component of antenatal care.

A platelet count of 50.000/µL or lower defines severe thrombocytopenia, a condition observed in only 1% of pregnancies yet associated with increased maternal and fetal morbidity, including postpartum hemorrhage, blood product transfusion, neonatal thrombocytopenia, and even neonatal intracranial hemorrhage [4,7]. Managing severe thrombocytopenia in pregnancy is particularly challenging, as it may arise from diverse etiologies with distinct pathophysiological mechanisms, therapeutic requirements, and prognostic implications for maternal-fetal health. Beyond common causes such as GT, ITP, preeclampsia, and HELLP syndrome, rare but life-threatening conditions such as thrombotic thrombocytopenic purpura (TTP) and atypical hemolytic uremic syndrome (aHUS) must also be considered [3,8]. Given this broad etiological spectrum, clinical management strategies and prognoses differ markedly, depending on the underlying cause. For instance, whereas GT requires only observation, ITP may necessitate immunomodulatory therapy, and conditions such as HELLP syndrome or TTP often require urgent delivery or plasma exchange to prevent severe maternal and fetal morbidity and mortality [8,9].

While mild and moderate thrombocytopenia during pregnancy (platelet count < 150.000 or <100.000/µL) has been extensively studied, data regarding severe thrombocytopenia in pregnancy remain limited. Despite its clinical importance, comprehensive information on the etiological spectrum and perinatal outcomes is still scarce. Therefore, this study aimed to determine the etiological distribution of severe thrombocytopenia in pregnant women managed at a tertiary perinatal center. Additionally, we sought to evaluate the demographic characteristics, treatment strategies, obstetric management, and perinatal outcomes associated with this high-risk condition and to investigate the correlations between minimum platelet levels and key clinical parameters.

## 2. Materials and Methods

### 2.1. Study Design and Participants

All consecutive pregnancies complicated by severe thrombocytopenia (platelet count 50,000/µL or lower) diagnosed at any point during pregnancy or at delivery and managed at our tertiary perinatology center between September 2020 and September 2025 were retrospectively reviewed and included in the study without sampling. The exclusion criteria were incomplete medical records, delivery at another institution, pancytopenia, multiple gestations, and cases in which thrombocytopenia was only diagnosed postpartum. A total of 241 patients met the study criteria, 38 of whom were excluded due to missing data on etiological diagnoses and perinatal outcomes. In total, 203 patients were included in the study. Participants were then stratified into three groups based on their minimum platelet count recorded during pregnancy: Group A (30,000 < platelet count ≤ 50,000/μL), Group B (10,000 < platelet count ≤ 30,000/μL), and Group C (platelet count ≤ 10,000/μL). Selection bias was minimized by including all eligible patients diagnosed within the study period, and diagnostic consistency was ensured by applying standardized hematological and obstetric criteria.

### 2.2. Definitions and Etiological Classifications

Complete blood count was performed using a D × H 800 analyzer (Beckman Coulter Inc., San Diego, CA, USA) with venous blood samples collected in ethylenediaminetetraacetate (EDTA) tubes. Thrombocytopenia was defined as a platelet count less than 150.000/µL. It was classified as mild when the platelet count was between 100.000 and 150.000/µL and moderate when it was between 50.000 and 100.000/µL. Severe thrombocytopenia was defined as a platelet count of 50,000/µL or lower [6]. Etiological diagnoses were established based on clinical presentation, laboratory findings, and well-defined diagnostic criteria. Gestational thrombocytopenia was diagnosed by exclusion of women with isolated thrombocytopenia presenting in the second or third trimester, with most cases having a platelet count more than 75.000/µL, with no prior history of low platelet counts and normalization of platelet levels within 1–2 months postpartum [3]. Immune thrombocytopenia (ITP) was diagnosed by exclusion according to international consensus criteria in women with isolated thrombocytopenia in the absence of other identifiable causes, particularly when thrombocytopenia was present before pregnancy or detected in the first trimester [10]. As part of the diagnostic evaluation, available records of autoimmune screening (including antinuclear antibody and antiphospholipid antibody tests) were reviewed, and cases with confirmed systemic autoimmune disease were classified according to their primary diagnosis rather than as ITP. The diagnosis of preeclampsia and HELLP (hemolysis, elevated liver enzymes, and low platelet count) syndrome was determined according to the 2019 criteria of the American College of Obstetricians and Gynecologists (ACOG) [11]. Preeclampsia was defined as new-onset hypertension (systolic blood pressure ≥ 140 mmHg or diastolic blood pressure ≥ 90 mmHg) after 20 weeks of gestation, accompanied by proteinuria or other maternal organ dysfunction [11]. HELLP syndrome, one of the most severe forms of preeclampsia, was diagnosed in the presence of microangiopathic hemolytic anemia, elevated liver transaminases, and thrombocytopenia [11]. Thrombotic thrombocytopenic purpura (TTP) was diagnosed based on the presence of microangiopathic hemolytic anemia, thrombocytopenia, and neurological symptoms, with or without renal impairment and fever [8]. Atypical hemolytic uremic syndrome (aHUS) was characterized by the triad of microangiopathic hemolytic anemia, thrombocytopenia, and acute kidney injury [8]. Pseudo-thrombocytopenia was confirmed by peripheral blood smear examination, demonstrating platelet clumping in EDTA-anticoagulated samples [3]. Other rare causes included inherited platelet disorders, disseminated intravascular coagulation (DIC) diagnosed according to the International Society for Thrombosis and Homeostasis scoring system using platelet count, prothrombin time, fibrin degradation products, fibrinogen level, and massive obstetric hemorrhage defined as blood loss > 2500 mL requiring massive blood transfusion [12,13]. All pregnancies complicated by severe thrombocytopenia received consultation opinions from hematologists.

### 2.3. Data Collection and Variables

Data were extracted from electronic medical records and included maternal demographic characteristics, such as age, gravidity, parity, body mass index (BMI), and history of thrombocytopenia before the current pregnancy. Obstetric variables included gestational age at diagnosis of thrombocytopenia, prepartum platelet count, minimum platelet count during pregnancy, postpartum platelet count, obstetric complications, and mode of delivery (spontaneous vaginal delivery or cesarean section). Laboratory parameters recorded included prepartum and postpartum hemoglobin levels, and the percentage decrease in hemoglobin levels from the prepartum to postpartum period. Treatment modalities for thrombocytopenia were documented, including platelet transfusion, corticosteroids, intravenous immunoglobulin (IVIG), plasmapheresis, and eculizumab, either as monotherapy or in combination. Perinatal outcomes assessed included gestational age at delivery, neonatal birth weight, neonatal sex, and Apgar scores. Etiological diagnoses were recorded for all participants.

### 2.4. Ethical Approval

The study protocol was reviewed and approved by the Institutional Ethics Committee (approval date and number: 2025/276). Given the retrospective nature of the study and the use of anonymized data, the requirement for informed consent was waived by the ethics committee in accordance with institutional guidelines and the Declaration of Helsinki.

### 2.5. Statistical Analysis

All statistical analyses were performed using IBM SPSS Version 24.0 (Armonk, NY, USA). Before comparing the variables, the distributions of the continuous variables were assessed for normality by examining histogram plots, evaluating kurtosis and skewness values, and performing the Kolmogorov–Smirnov and Shapiro–Wilk tests. Normally distributed continuous variables were expressed as mean ± standard deviation and compared using a one-way ANOVA test. Games–Howell post hoc tests were used for pairwise comparisons. Variables that were not normally distributed were reported as median (minimum-maximum) and were compared using the Kruskal–Wallis H test with post hoc pairwise Mann–Whitney U tests. A *p*-value of <0.05 was considered statistically significant. Categorical variables were expressed as percentages (%), and comparisons were performed using the chi-square test. For group-to-group comparisons, pairwise chi-square tests with the Bonferroni correction were used to identify which groups differed significantly. According to the Bonferroni correction, a *p*-value of <0.016 was considered statistically significant. Adjusted residuals were examined to determine variables that were statistically significant in each group. As the study population consisted of all eligible cases identified within the specified time frame, no prior sample size calculation was required.

## 3. Results

This retrospective cohort study included 203 pregnant women with severe thrombocytopenia. The participants were stratified into three groups based on their minimum platelet counts: Group A (30,000 < platelet count ≤ 50,000/μL, *n* = 123, 60.6%), Group B (10,000 < platelet count ≤ 30,000/μL, *n* = 54, 26.6%), and Group C (≤10,000/μL, *n* = 26, 12.8%). The demographic characteristics of the pregnant women and treatment modalities are presented in Table 1. The mean maternal ages of pregnant women were 30.1 (±6.14), 29.5 (±6.37), and 29.7 (±6.11) years, respectively, and no statistically significant difference was observed in terms of this parameter (F_2,200_ = 0.166, *p* = 0.847). The median gestational ages at diagnosis in the study groups were 34 (8–41.2), 33.4 (12–40.5), and 32.7 (15.4–39) weeks, respectively, with no statistically significant difference (*p* = 0.331). Comparison of BMI among the patients revealed no statistically significant difference (F_2,200_ = 0.733, *p* = 0.482). No statistically significant differences were observed among the obstetric histories of the participants, including gravida, parity, and abortion (*p* = 0.923, *p* = 0.653, *p* = 0.329, respectively).

The mean minimum platelet counts of the study groups were 42,552 (±6327), 21,666 (±5449), and 6792 (±2310), respectively, which revealed that the minimum platelet count was significantly lower in Group C than in groups A and B (Welch’s F_2,101_ = 1197, *p* = 0.001). While prepartum platelet levels were significantly lower in Group C than in Groups A and B (*p* = 0.001), postpartum platelet levels were lower in Groups B and C than in Group A (*p* = 0.001). Statistically significant differences were observed in the presence of thrombocytopenia before pregnancy between the study groups (*p* = 0.001). Pairwise comparisons indicated that while the difference between Groups A and B was not statistically significant, the rate of thrombocytopenia before pregnancy was significantly lower in Groups A and B than in Group C (*p* = 0.001).

Evaluation of treatment modalities for thrombocytopenia revealed a statistically significant difference among the study groups with respect to these parameters (*p* = 0.001). Examination of adjusted residuals indicated that the proportion of patients who did not require treatment was significantly higher in Group A than in Groups B and C (adjusted residual = 2.2). Requirement for complex treatment modalities, including steroid + IVIG + platelet transfusion (adjusted residual = 5.6), steroid + platelet transfusion (adjusted residual = 3.6) and plasmapheresis + platelet transfusion (adjusted residual = 3.5) was more pronounced in Group C than in Group A and B. Comparison of Group B and C in with respect to treatment requirement revealed that the proportion of patients not requiring treatment was higher in Group B (adjusted residual = 3.2).

The etiological causes of severe maternal thrombocytopenia and maternal and neonatal outcomes of the study population are shown in Table 2. The median gestational ages at delivery in the study groups were 37 (23.3–41.3), 37.2 (24–41), and 36.9 (29–39.4) weeks, respectively, and no statistically significant differences were observed in terms of this parameter (*p* = 0.587). The mean birth weights were 2477 (±1086), 2605 (±799), and 2547 (±968) in groups A, B, and C, respectively. Comparison of this parameter revealed no statistically significant difference (Welch’s F_2,81.3_ = 1.68, *p* = 0.191). Similarly, sex and mode of delivery did not differ significantly (*p* = 0.754, *p* = 0.083, respectively). The occurrence of obstetric complications, including placental abruption, fetal distress, emergency cesarean section, and postpartum hemorrhage, was comparable between the study groups (*p* = 0.070). Among hematological parameters, a significant difference was observed in prepartum hemoglobin levels (Welch’s F_2,79.3_ = 7.91, *p* = 0.001), whereas postpartum hemoglobin levels (F_2,200_ = 1.21, *p* = 0.254) and the rate of decrease in Hgb levels were not statistically significant (Welch’s F_2,73.6_ = 1.64, *p* = 0.200). Games–Howell post hoc pairwise comparisons of prepartum hemoglobin levels indicated that a statistically significant difference was observed only between groups A and C (*p* = 0.001), whereas no significant differences were found between the other group pairs (*p* = 0.674, *p* = 0.130). Etiological causes of severe maternal thrombocytopenia were significantly different among the study groups (*p* = 0.001). Evaluation of adjusted residuals applied to determine which etiological causes differed significantly among the study groups revealed that the proportion of pseudo-thrombocytopenia was comparable across all groups (adjusted residual = 0.3). The most common cause in the overall cohort was HELLP syndrome, accounting for 74 cases (36.5%), followed by immune thrombocytopenia (ITP) in 47 cases (23.2%), and pseudo-thrombocytopenia in 39 cases (19.2%). Obstetrical causes, including HELLP syndrome (*n* = 50, 40.7%) (adjusted residual = 2.3) and gestational thrombocytopenia (*n* = 15, 12.2%) (adjusted residual = 3.2), were significantly more common in Group A compared with Groups B and C. In contrast, non-obstetrical causes, including ITP and TTP, were more pronounced in Group C compared with Group A (adjusted residual = 4.5), whereas gestational thrombocytopenia was absent, and HELLP syndrome was encountered in only one case (3.8%). These findings are consistent with the observation that, as the severity of thrombocytopenia increases, the likelihood of an underlying non-obstetric cause also increases.

Correlation analyses performed in the study population between the minimum maternal platelet levels and the rate of decrease in Hgb levels, GA at delivery, GA at diagnosis, and birth weight are presented in Table 3. There was no statistically significant correlation between minimum maternal platelet levels and these parameters. (r = −0.330, *p* = 0.642, r = −0.130, *p* = 0.0063, r = 0.120, *p* = 0.088, r = −0.087, *p* = 0.219).

## 4. Discussion

This retrospective cohort study of pregnant women with severe thrombocytopenia, managed at a tertiary perinatology center, revealed a distinct etiological distribution that varied significantly according to the degree of platelet depletion. Although HELLP syndrome emerged as the predominant cause in the overall cohort, ITP was the leading diagnosis among the most severe cases. The etiological distribution observed in this cohort warrants careful consideration in the context of the existing literature. Our finding that HELLP syndrome accounted for more than one-third of cases is substantially higher than the proportion reported in previous studies of thrombocytopenia in pregnancy, where hypertensive disorders typically represent approximately 8–12% of cases [14,15]. In a prior study investigating patients with a platelet count less than 100.000/µL, the proportion of hypertensive disorders increased slightly to 22% [6]. In studies focusing on cases with a platelet count below 50.000/µL, hypertensive disorders accounted for 42.5% of cases, consistent with our findings [7]. In a more recent study including patients with severe thrombocytopenia (<50.000/µL), hypertensive disorders accounted for 66.7% of all etiologies [16]. These findings collectively suggest that as the platelet count decreases, hypertensive disorders become increasingly frequent as a cause of thrombocytopenia. Another potential explanation for the high rate of hypertensive disorders in our cohort may be the referral pattern to our tertiary perinatology center, which receives a disproportionate number of high-risk pregnancies complicated by preeclampsia with severe features.

The observation that HELLP syndrome predominated in Group A (30,000 < platelet count ≤ 50,000/μL) and Group B (10,000 < platelet count ≤ 30,000/μL), while becoming markedly less frequent in the most severe category (Group C = platelet count ≤ 10,000/μL) is consistent with the pathophysiology of this condition, which typically manifests with platelet counts in the range of 20,000–100,000/µL rather than profound thrombocytopenia, as it represents an acute consumptive process that generally resolves rapidly following delivery [17]. Conversely, the predominance of ITP in the most severe thrombocytopenia group aligns with the natural history of this autoimmune disorder, which can lead to profound platelet depletion [9]. This finding aligns with the work of Zhou et al., who demonstrated that severe primary autoimmune thrombocytopenia during pregnancy is frequently associated with platelet counts below 30,000/µL [14]. The significantly higher proportion of patients with pre-existing thrombocytopenia in Group C (46.2%) further supports the contribution of chronic immune-mediated or inherited platelet disorders in the most severe cases. In line with previous studies, in our cohort, the lower the platelet count, the less likely the diagnosis was of gestational thrombocytopenia, and notably, no cases of gestational thrombocytopenia were observed in group C [16]. The relatively high frequency of pseudo-thrombocytopenia across all severity groups, representing nearly one-fifth of cases, emphasizes the critical importance of confirmatory peripheral blood smear examination before initiating potentially unnecessary interventions [18].

The divergent etiological patterns observed across severity groups likely reflect different mechanisms of platelet depletion. Obstetrical causes such as HELLP syndrome represent acute consumptive processes characterized by endothelial dysfunction, platelet activation, and accelerated clearance in the context of severe preeclampsia [2]. Platelet reduction in HELLP syndrome is generally self-limiting and resolves rapidly following delivery, which explains the lower prevalence of this diagnosis in patients with the most profound thrombocytopenia [19]. In contrast, non-obstetrical causes such as ITP are characterized by antibody-mediated platelet destruction and impaired megakaryopoiesis, leading to persistent and often more profound thrombocytopenia that may present early in pregnancy and persist throughout gestation [20]. Similarly, TTP represents a thrombotic microangiopathy with severe platelet consumption that frequently produces profound thrombocytopenia and requires urgent therapeutic intervention [21]. These etiological differences have important clinical implications as each underlying cause of severe thrombocytopenia requires different antenatal surveillance and management strategies. HELLP syndrome necessitates expeditious delivery once maternal or fetal compromise is evident, whereas ITP may be managed expectantly with medical therapy to optimize platelet count for safe delivery [3,9]. In our study, the absence of significant differences in gestational age at delivery, birth weight, and neonatal outcomes across severity groups suggests that appropriate etiological diagnosis and tailored management may mitigate the potential adverse effects of severe thrombocytopenia. Similar to the findings of Zhou et al., a total of 83.7% of the overall cohort underwent cesarean section due to obstetric indications or maternal request associated with maternal anxiety, fear of labor pain, or a previous negative birth experience [14]. However, the rate of cesarean delivery did not differ across severity groups, suggesting that with appropriate management of the thrombocytopenia, the timing and mode of delivery should be determined by obstetric indications rather than platelet count alone [3,22].

In our cohort, we observed no significant difference in obstetric complications, including postpartum hemorrhage, across the severity of thrombocytopenia, consistent with previous studies [14,22]. Postpartum hemoglobin values and the rate of decrease in hemoglobin levels were also comparable among the groups, further suggesting no significant difference in hemorrhagic complications. However, the significantly lower prepartum hemoglobin levels in Group C compared with Group A may reflect the chronic nature of the underlying disorder rather than an acute peripartum hemorrhagic event.

The therapeutic patterns observed in this study reflect contemporary clinical practice in the management of severe gestational thrombocytopenia. It is rational and expected that treatment becomes more aggressive with greater severity of thrombocytopenia, considering the significantly heightened risk of hemorrhage at critically low platelet count [4]. In our cohort, while 79.7% of patients in group A required no treatment, 73.1% of patients in group C received platelet transfusion, steroid, intravenous immunoglobulin (IVIG), plasmapheresis, or a combination of these therapies. The frequent use of combination therapy in the most severe group, predominantly composed of ITP cases, including corticosteroids, intravenous immunoglobulin, and platelet transfusion, underscores the challenges of rapidly increasing platelet counts in refractory cases to reduce the risk of hemorrhage [9,22]. The significantly higher proportion of untreated patients in Group A compared to Groups B and C, along with the observation that nearly 64.7% of the overall cohort required no specific treatment, reflects the benign nature of many cases, particularly those attributable to gestational thrombocytopenia or pseudo-thrombocytopenia [4]. The requirement for plasmapheresis combined with platelet transfusion in Group C likely represents cases of thrombotic microangiopathies, such as TTP, highlighting the importance of recognizing these rare but life-threatening conditions that require distinct therapeutic approaches [23]. The observation that even within Group C, 26.9% of patients required no treatment suggests that therapeutic decision-making should be guided primarily by the etiology and overall clinical context rather than platelet count alone.

The absence of statistically significant correlations between minimum platelet count and key clinical parameters, including gestational age at delivery, birth weight, gestational age at diagnosis, and the rate of hemoglobin decline, was remarkable. This finding suggests that platelet count alone may not be a reliable predictor of obstetric or hemorrhagic outcomes and that the underlying etiology and associated comorbidities may be more important determinants of perinatal outcomes [16,22]. This observation has important implications for clinical decision-making, as it argues against rigid platelet count thresholds for intervention, and supports a more nuanced, etiology-based approach to management [15].

This study has several methodological strengths that enhance the validity and generalizability of its findings. The cohort was recruited from a tertiary perinatology center with extensive experience in managing high-risk pregnancies and access to advanced diagnostic evaluation and specialized management. The relatively large sample size of 203 cases over a five-year period provides adequate statistical power to detect clinically meaningful differences. Stratifying patients into three severity groups based on minimum platelet count allowed for a granular analysis of the relationship between severity and etiology. The use of rigorous statistical methods, including appropriate tests for normality, post hoc pairwise comparisons with the Bonferroni correction, and examination of adjusted residuals, strengthened the validity of the findings. However, this study has several limitations. The retrospective design precludes definitive causal inferences and may be subject to selection bias, as patients managed at tertiary centers may not be representative of the broader population of pregnant women with thrombocytopenia. The absence of long-term maternal and neonatal follow-up data limited our ability to assess delayed complications. Additionally, the relatively small number of cases in certain etiological categories, particularly TTP and other rare causes, precluded detailed subgroup analyses of these clinically important conditions.

## 5. Conclusions

Severe thrombocytopenia in pregnancy represents a heterogeneous condition with distinct etiological patterns that vary according to the degree of platelet depletion, underscoring the need for individualized diagnostic and therapeutic strategies to optimize maternal and fetal outcomes. The marked etiological heterogeneity observed across severity groups highlights the importance of comprehensive evaluation, rather than relying solely on platelet count for risk stratification. Clinicians should maintain a high index of suspicion for non-obstetrical causes such as ITP in cases of profound thrombocytopenia, particularly when thrombocytopenia predates pregnancy or presents in the first trimester. Conversely, the acute onset of thrombocytopenia in conjunction with hypertension should trigger consideration of obstetric conditions such as preeclampsia and HELLP syndrome. The favorable perinatal outcomes observed across all severity groups, even among patients with profound thrombocytopenia, suggest that when appropriately managed at specialized centers, severe thrombocytopenia does not invariably confer poor prognosis. Future prospective studies are warranted to develop and validate predictive models that integrate both platelet count and etiological factors to guide individualized clinical decision-making.

## Figures and Tables

**Table 1:** The demographic characteristics and the perinatal outcomes of pregnant women diagnosed with thrombocytopenia.

		Platelet Counts × 10^9^/L				
		Group A (30,000 < Platelet Count ≤ 50,000/μL) (*n* = 123)	Group B (10,000 < Platelet Count ≤ 30,000/μL) (*n* = 54)	Group C (≤10,000/μL) (*n* = 26)	Total (*n* = 203)	*p* Values ^a^
Age (years) ^b^		30.1 (±6.14)	29.5 (±6.37)	29.7 (±6.11)	29.9 (±6.17)	0.847
Gravida (n) ^c^		2 (1–7)	2 (1–7)	2 (1–8)	2 (1–8)	0.923
Parity (n) ^c^		1 (0–5)	1 (0–6)	1 (0–4)	1 (0–6)	0.653
Abortion (n) ^c^		0 (0–3)	0 (0–3)	0 (0–7)	0 (0–7)	0.329
BMI (kg/m^2^) ^b^		25.8 (±2.17)	26.2 (±3.92)	25.4 (±1.83)	25.9 (±2.71)	0.482
GA at diagnosis ^c^		34 (8–41.2)	33.4 (12–40.5)	32.7 (15.4–39)	33.9 (8–41.2)	0.331
Thrombocytopenia before pregnancy ^d^		9 (7.3%)	7 (13%)	12 (46.2%)	28 (13.8%)	0.001
Prepartum platelet ^c^		84 (34–306)	51 (18–327)	31 (5–324)	74 (5–327)	0.001
Minimum platelet ^c^		42,552 (±6327)	21,666 (±5449)	6792 (±2310)	32,416 (±14,512)	0.001
Postpartum platelet ^c^		67 (33–275)	47.5 (21–71)	53 (4–570)	62 (4–71)	0.001
Treatments						0.001
	None	98 (79.7%)	25 (46.3%)	7 (26.9%)	130 (64.7%)	
	Platelet transfusion	19 (15.4%)	14 (25.9%)	1 (3.8%)	34 (16.7%)	
	Steroid + IVIG + PT	0 (0%)	3 (5.6%)	8 (30.8%)	11 (5.4%)	
	Steroid + PT	1 (0.8%)	5 (9.3%)	6 (23.1%)	12 (5.9%)	
	IVIG	1 (0.8%)	1 (1.9%)	1 (3.8%)	3 (1.5%)	
	Steroid + IVIG	1 (0.8%)	2 (3.7%)	0 (0%)	3 (1.5%)	
	Plasmapheresis + PT	2 (1.6%)	4 (7.4%)	2 (7.7%)	8 (3.9%)	
	Plasmapheresis + EC	1 (0.8%)	0 (0%)	0 (0%)	1 (0.5%)	
	IVIG + PT	0 (0%)	0 (0%)	1 (3.8%)	1 (0.5%)	

BMI: body mass index; GA: gestational age. ^a^ Level of significance *p* < 0.05. ^b^ Continuous variables that were normally distributed are expressed as mean ± standard deviation and were compared using the One-way ANOVA test. ^c^ Data that were not normally distributed are expressed as median (minimum-maximum) and were compared using Kruskal–Wallis H test with post hoc Mann–Whitney U test. ^d^ Categorical variables, presented as percentage (%) and count (n), were compared using the Chi-Square test, and for pairwise comparison, the Pairwise Chi-Square test and adjusted standardized residuals were used.

**Table 2:** Etiological causes and maternal neonatal outcomes of the study population.

		Platelet Counts × 10^9^/L				
		Group A (30,000 < Platelet Count ≤ 50,000/μL) (*n* = 123)	Group B (10,000 < Platelet Count ≤ 30,000/μL) (*n* = 54)	Group C (≤10,000/μL) (*n* = 26)	Total (*n* = 203)	*p* Values ^a^
GA at delivery (weeks) ^c^		37 (23.3–41.3)	37.2 (24–41)	36.9 (29–39.4)	37 (23.3–41.3)	0.587
Birth weight (gr) ^b^		2477 (±1086)	2605 (±799)	2762 (±611)	2547 (±968)	0.191
Sex ^d^						0.754
	Female	52 (42.3%)	23 (42.6%)	9 (34.6%)	84 (41.4%)	
	Male	71 (57.7%)	31 (57.4%)	17 (65.4%)	119 (58.6%9	
Prepartum Hgb ^b^		11.6 (±1.46)	11.4 (±1.89)	10.8 (±0.85)	11.4 (±1.55)	0.001
Postpartum Hgb ^b^		9.9 (±1.49)	10.1 (±1.70)	9.45 (±1.68)	9.92 (±1.58)	0.254
Decrease rate in Hgb levels (%) ^b^		15.1 (±9.31)	17.2 (±6.96)	14.9 (±6.48)	15.7 (±8.44)	0.200
Presence of obstetrics complication ^d^		65 (52.8%)	31 (57.4%)	8 (30.2%)	104 (51.2%)	0.070
Mode of Delivery ^d^						0.083
	SVD	16 (13%)	9 (16.7%)	8 (30.8%)	33 (16.3%)	
	CS	107 (87%)	45 (83.3%)	18 (69.2%)	170 (83.7%)	
Etiology ^d^						0.001
	Pseudo-thrombocytopenia	25 (20.3%)	9 (16.7%)	5 (19.2%)	39 (19.2%)	
	Gestational thrombocytopenia	15 (12.2%)	0 (0%)	0 (0%)	15 (7.4%)	
	HELLP	50 (40.7%)	13 (42.6%)	1 (3.8%)	74 (36.5%)	
	ITP	18 (14.6%)	14 (25.9%)	15 (57.7%)	47 (23.2%)	
	TTP	1 (0.8%)	2 (3.7%)	4 (15.4%)	7 (3.4%)	
	aHUS	1 (0.8%)	1 (1.9%)	0 (0%)	2 (1%)	
	Others	9 (7.3%)	4 (7.4%)	0 (0%)	13 (6.4%)	

SVD: spontaneous vaginal delivery; CS: cesarean section; GA: gestational age; Hgb: hemoglobin; ITP: immune thrombocytopenia; TTP: thrombotic thrombocytopenic purpura; aHUS: atypical hemolytic uremic syndrome; Others: Bernard–Soulier Syndrome, Glanzman thrombocytopenia, pancytopenia, Ig A nephropathy, megaloblastic anemia. ^a^ Level of significance *p* < 0.05. ^b^ Continuous variables that were normally distributed are expressed as mean ± standard deviation and were compared using the One-way ANOVA test with Games–Howell post hoc tests for pairwise comparison. ^c^ Data that were not normally distributed are expressed as median (minimum-maximum) and were compared using the Kruskal–Wallis H test. ^d^ Categorical variables, presented as percentage (%) and count (n), were compared using the Chi-Square test, and for pairwise comparison, Pairwise Chi-Square test and adjusted standardized residuals were used.

**Table 3:** Correlation analyses between minimum platelet levels and clinical parameters in the study population.

Minimum Platelet		
	r	*p* ^a^
Decrease rate in Hgb levels (%)	−0.330	0.642
GA at delivery (weeks)	−0.130	0.063
GA at diagnosis	0.120	0.088
Birth weight	−0.087	0.219

GA: gestational age. r = Pearson’s correlation. ^a^ Level of significance *p* < 0.05.

## Data Availability

The data that support the findings of this study are available upon reasonable request from the corresponding author.
